# Case of Monstrosity

**Published:** 1874-04

**Authors:** F. E. English

**Affiliations:** Altoona, Polk Co., Iowa


ARTICLE IV.—*Case of Monstrosity.* By F. E. ENGLISH, M.D., Altoona, Polk Co., Iowa.

The following notes were handed me by my father, R. George English, M.D., of Des Moines, Iowa, and as the case is one the like of which is seldom met with, I concluded to submit it for the consideration of the readers of the JOURNAL, if thought worthy of a place therein.

"Case. Called at 9 o'clock P. M., Nov. 4th, 1873, to Mrs. R. L., aged 24 years; fine physical organization; rather above medium size, and in good health. Primapara; said her term of gestation had not expired by about two weeks; had had premonitions of labor, however, for twenty-four hours, and at present decided uterine contractions declared the fact that her delivery could not be far in the future. A very noticeable feature in the case was the enormous distension of the abdomen; digital examination revealed a capacious pelvis, complete dilatation of the os tincæ, and at every uterine contraction the membranes descended unusually low, and yet at no time previous to their rupture could a presentation be detected. The rupture was followed by a most extraordinary flow of liquor amnii, not less than ten certainly, and perhaps as much as twelve, quarts, followed by a rapid decrease in the size of the abdomen, soon after which a presentation was detected, but what was it? Vertex, breach, shoulder, elbow, knee, or footling, for my life I could make no satisfactory diagnosis. The old homely adage occurred to me, —'Feeling is believing, but seeing is the naked truth,' but I was scarcely able to form an intelligent belief; diagnosis failed me. The digit came in contact with an aperture scarcely large enough to admit the point of the finger, surrounded by irregular elevations and depressions upon a bony surface. No such presentation had ever occurred in the course of my somewhat extensive practice, covering a period of more than a score of years, and I was forced to admit (to myself, of course,) that I did not know what even to reasonably anticipate; but having full confidence in the ability of my patient to meet all the requirements of the labor, even to its completion, I could well afford to wait, and after neither a very hard nor yet a protracted labor my patient was delivered of a female baby, well developed in every respect, except the head, neck, and upper portion of the spinal column. The mouth was small and pouting, the eyes were well enough developed, but above the supra-orbital plates there were no cranial bones; the ears were well formed and in their normal positions, a belt of scalp extending, as it were, across the upper end of the body and covered with black hair which curled beautifully around each ear. As the neck was entirely wanting, the sternum extended to near the chin, which seemed to rest upon its superior extremity, and formed a point farther than any other from the lower extremity of the body. From the supra-dorsal (the cervical vertebræ were wanting) to about the middle of the lumbar region of the spine, that column presented three instead of one ridge or row of processes, the central having nearly the normal appearance, while the right and left lateral were irregularly curved outward and upward as the body lay on its anterior aspect. Situated at the superior extremity of this triple column, and upon its posterior aspect, was a small quantity of brain substance, resembling the cerebrum. The cerebellum and medulla oblongata were not discoverable."

This was the condition in which nature ushered into the world a human being, as is seen from the above notes. All the contents of the encephalon were entirely wanting, except a rudimentary cerebrum; and that the fœtus was living to within a short time of its birth is evident from its almost perfect bodily development, as well as the evidence of motion felt by the mother a few moments prior to its birth. The thoracic and abdominal viscera were not examined, but to all external appearances were well developed and perfect.

My object in making a report of this case, in the first place, is, its rare occurrence, and second, to invite the views of others in regard to this class of cases. One point I wish to arrive at is, upon what physiological principle can the want of development of so important a part of the body as the head, be accounted for? or can it be accounted for physiologically at all? I am aware that the members of our profession are loth to believe, and in fact almost entirely discredit, the influences that are commonly considered as the cause of the so-called "birth marks." Are we authorized to entertain the belief that there are scientific causes why the process of development should be arrested in some part of the fœtus in utero and the full and perfect work completed in the remaining parts? In the case before us there seems to have been an overwork performed in the one part, (the triple row of spinal processes), and an entire absence of others, (the cranial bones, with a part of their contents.) Have the influences brought to bear on the mind during the period of gestation anything definite to do in controlling the development of the fœtus in utero? If this be conceded, another very important query arises, What class of emotions will arrest or retard the perfect formation of an arm, leg, finger, or toe? and what to prevent the development of brain, bone, etc., etc.? or what to produce a supplemental member, as a finger or toe? Have these queries a satisfactory solution in science, if we but had the key?

